# Sex-specific effects of calving season on joint health and biomarkers in Montana ranchers

**DOI:** 10.1186/s12891-022-05979-2

**Published:** 2023-01-31

**Authors:** Matthew A. Thompson, Stephen A. Martin, Brady D. Hislop, Roubie Younkin, Tara M. Andrews, Kaleena Miller, Ronald K. June, Erik S. Adams

**Affiliations:** 1 Department of Chemical & Biological Engineering, Montana State University, Bozeman, MT USA; 2 Center for American Indian and Rural Health Equity, Translational Biomarkers Core Laboratory, Montana State University, Bozeman, MT USA; 3 Department of Mechanical & Industrial Engineering, Montana State University, PO Box 173800, Bozeman, MT 59717-3800 USA; 4 MSU Extension Office, Montana State University, Bozeman, MT USA; 5 School of Medicine, Montana WWAMI, University of Washington, Seattle, WA USA

**Keywords:** Biomarkers, Osteoarthritis, Sex differences, Inflammation, Joint pain, Ranching, Calving season

## Abstract

**Background:**

Agricultural workers have a higher incidence of osteoarthritis (OA), but the etiology behind this phenomenon is unclear. Calving season, which occurs in mid- to late-winter for ranchers, includes physical conditions that may elevate OA risk. Our primary aim was to determine whether OA biomarkers are elevated at the peak of calving season compared to pre-season, and to compare these data with joint health survey information from the subjects. Our secondary aim was to detect biomarker differences between male and female ranchers.

**Methods:**

During collection periods before and during calving season, male (*n* = 28) and female (*n* = 10) ranchers completed joint health surveys and provided samples of blood, urine, and saliva for biomarker analysis. Statistical analyses examined associations between mean biomarker levels and survey predictors. Ensemble cluster analysis identified groups having unique biomarker profiles.

**Results:**

The number of calvings performed by each rancher positively correlated with plasma IL-6, serum hyaluronic acid (HA) and urinary CTX-I. Thiobarbituric acid reactive substances (TBARS), a marker of oxidative stress, was significantly higher during calving season than pre-season and was also correlated with ranchers having more months per year of joint pain. We found evidence of sexual dimorphism in the biomarkers among the ranchers, with leptin being elevated and matrix metalloproteinase-3 diminished in female ranchers. The opposite was detected in males. WOMAC score was positively associated with multiple biomarkers: IL-6, IL-2, HA, leptin, C2C, asymmetric dimethylarginine, and CTX-I. These biomarkers represent enzymatic degradation, inflammation, products of joint destruction, and OA severity.

**Conclusions:**

The positive association between number of calvings performed by each rancher (workload) and both inflammatory and joint tissue catabolism biomarkers establishes that calving season is a risk factor for OA in Montana ranchers. Consistent with the literature, we found important sex differences in OA biomarkers, with female ranchers showing elevated leptin, whereas males showed elevated MMP-3.

**Supplementary Information:**

The online version contains supplementary material available at 10.1186/s12891-022-05979-2.

## Background

Osteoarthritis (OA) is a chronic, degenerative joint disease estimated to affect at least 31 million US adults [1]. Advances in the understanding and treatment of OA are needed for those suffering from this disease. Agricultural workers experience a higher prevalence of OA compared to other occupations with similar levels of physical activity [2–5]. A large population-based study showed the relative risk (RR) of hospitalization for knee OA in men, compared to sedentary occupations, was 3.78 for farmers, 2.08 for dock workers, and 1.78 for fishermen [6]. A Danish population registry study found that male farmers had an incidence rate for hip OA of 157.7 cases per 100,000 person-years, compared to 89.9 for construction workers. For women, the contrast was less marked, at 103.7 for farmers and 93.8 for construction workers [7].

.

For farmers, disability from OA may affect the economic health of their farm/ranch as well as the food supply. We previously found a correlation between higher WOMAC (Western Ontario and McMaster Universities Arthritis Index) pain scores and both worsening self-reported financial health of the operation and diminished self-reported ability to perform work [8].

Our focus groups with Montana cattle ranchers indicated that calving season is particularly strenuous, featuring several OA risk factors, including sleep deprivation, increased joint loads, cold weather exposure, and possibly a diet higher in convenience foods [8]. The present follow-up case–control study sought to determine whether OA biomarkers in blood and urine are elevated at the peak of calving season (“in-season”), compared to pre-season. A more thorough understanding of the factors influencing joint health during calving season and the relationships between OA biomarkers and joint health may inform risk reduction and disease management approaches in agricultural workers and possibly in other physically-demanding occupations.

Historically, OA was seen as a cartilage “wear and tear” phenomenon, but it is now appreciated that its etiology is complex. Systemic inflammation [9], local inflammatory factors and free radicals [10], enzymatic destruction of cartilage extracellular matrix components and subchondral bone [11, 12], resident stem cell dysfunction [13, 14], production of adipokines by fat [15, 16], aging [17, 18] and previous joint trauma [19] are all implicated.

Diagnosis of OA is currently based on radiographs and clinical symptomology (e.g., joint pain and functional impairment), but more recently the field has identified biochemical biomarkers in the blood, urine, and synovial fluid that can be utilized to detect OA, inform prognosis and monitor disease progression and response to treatment [20–24].

There are as yet no clinically validated biomarkers to detect OA or assess disease severity.

Both the epidemiology and pathophysiology of OA demonstrate sexual dimorphism. Women have a higher relative risk (RR) of knee and hand OA [25–27] and display a more rapid progression of cartilage damage in the knee [28]. Women with knee OA demonstrate gait alterations not evident in their male counterparts [29]. Cluster analysis of data from the Foundation for the National Institutes of Health Biomarkers Consortium show sexual dimorphism among groups [23]. Additionally, there was greater separation of clusters of biomarker results by sex than by progressor/non-progressor status. The sexual dimorphism was sufficiently conclusive that the authors argued for sex-stratified studies in knee OA [23]. Sexual dimorphism is seen in articular cartilage turnover biomarkers in early knee OA; pre-menopausal women show elevated levels of both serum collagen oligomeric matrix protein (COMP) and urine C-terminal telopeptide of collagen type II (CTX-II) than controls, whereas men show no such association [30]. There may also be sexual dimorphism with regard to leptin levels in OA [31]. Furthermore, bone turnover biomarkers were lower in pre-menopausal women than controls, and again men did not show this association [30].

A potential classification for OA biomarkers may be those that are responsible for the pathogenesis of OA and those that become elevated as a consequence of joint damage. The former category includes inflammatory cytokines, markers of tissue peroxidation, and enzymes that degrade bone and cartilage matrix; the latter includes molecular fragments obtained from bone and cartilage enzymatic degradation. Using biomarkers from both categories, the primary aim of this study is to assess heightened inflammation and evidence of joint damage from participation in calving season. We hypothesize that farmers and ranchers experience dysregulation in standard OA biomarkers during calving season that would point to an increased risk of OA. Profiling similar biomarker and joint responses identified groups of ranchers that were disposed to particular OA outcomes. Comparing plasma, serum, and urine biomarker levels against survey-determined predictors of joint health enabled characterization of relationships between calving season and biomarkers important to OA pathology. Our secondary aim was to detect sex-specific responses within this sample of ranchers, hypothesizing that differences between the sexes (dimorphism) would be seen in the directions and magnitudes of biomarker changes.

## Methods

### Participant recruitment

The study was approved by the Montana State University (MSU) Institutional Review Board (EA043019), and all studies were carried out in accordance with relevant guidelines and regulations. All participants signed informed consent. Participant recruitment was conducted by MSU Extension county agents between May 1 and October 15, 2019. MSU Extension has agents in each of 56 Montana counties and the 7 tribal reservations. Participants were from Valley, Custer, Madison, and Jefferson counties in Montana, and all participants were ranchers whose operations included calving. Inclusion and exclusion criteria are shown in Table 1. The decision to include those using chewing tobacco but not smoking was made because of the ubiquity of the former among male ranchers. Participants were met either at their ranch or in a nearby town, and each rancher completed surveys and collection of blood, urine, and saliva samples prior to and in the middle of calving season.

**Table 1:** Inclusion and exclusion criteria

Inclusion	Exclusion
Calving operation produces at least 120 calves/season	Diabetes
Age 30–70	Smoking in the past year (chewing tobacco OK)
	Current cancer or in remission less than 3 years
	Autoimmune disease
	Inflammatory condition (e.g., gout, Crohn’s disease, ulcerative colitis, rheumatoid arthritis)
	Corticosteroids in past 3 months
	Bisphosphonate use

### Biospecimen collection and biomarker analysis

**Fig. 1:**
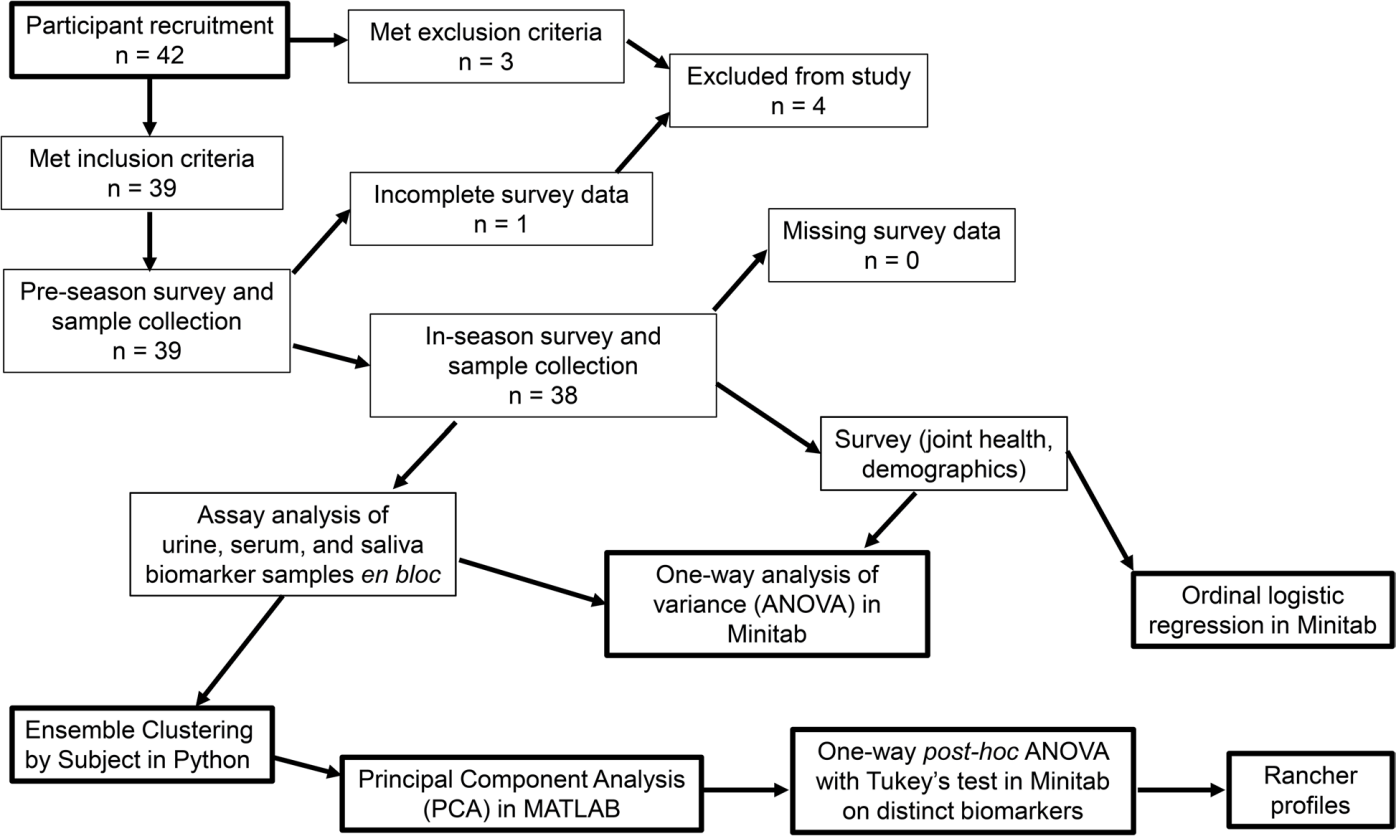
Study design to examine biomarkers and OA symptoms in ranchers before and during calving season

Blood was collected by routine venipuncture into serum separator, heparin, and EDTA-containing Vacutainer tubes. All biospecimen samples were labeled and refrigerated after collection, either using the onboard refrigerator in MSU’s Health, Education, and Research Bus (HERB), a mobile laboratory outfitted for field research, or with a portable refrigerator. Centrifugation of blood samples occurred within 8 h of collection. Saliva was collected as a passive drool sample and urine in a urinalysis cup. Aliquoted samples were stored at -80 °C until analyses. Figure 1 displays a flow chart illustrating study design and analysis steps. Biomarkers related to OA were assayed using commercially available kits (Table 2). Urine creatinine concentration was used to normalize urine biomarker concentrations after assaying and was not evaluated as a response.

**Table 2:** Osteoarthritis biomarkers assayed

Biomarker, sample type	Biomarker name	Role in OA pathogenesis	Reference	Assay method, dilution ratio
**Inflammatory Biomarkers**				
IL-1ra, plasma heparin	Interleukin-1 receptor antagonist	Blocks IL-1 but is elevated in OA	[32]	R&D Systems Luminex FCSTM03, 1:3
IL-2, plasma heparin	Interleukin-2	Anti-inflammatory, immunoregulatory cytokine	[22]	R&D Systems Luminex FCSTM09, 1:2
IL-4, plasma heparin	Interleukin-4	Anti-inflammatory, immunoregulatory cytokine	[22]	R&D Systems Luminex FCSTM09, 1:2
IL-6, plasma heparin	Interleukin-6	Pro-inflammatory cytokine, macrophage-derived	[33, 34]	R&D Systems Luminex FCSTM09, 1:2
IL-8, plasma heparin	Interleukin-8	Chemotactic factor	[35]	R&D Systems Luminex FCSTM09, 1:2
MCP-1, plasma heparin	Monocyte chemoattractant protein-1, aka C–C motif ligand 2 (CCL2)	Monocyte and memory T cell recruitment	[36]	R&D Systems Luminex FCSTM03, 1:3
HA, Plasma EDTA	Hyaluronic acid or hyaluronan	Levels positively associated with OA severity, pain, progression & duration	[37]	Biovision E4626-100 1:100
CRP, plasma heparin	C-reactive protein	Marker of systemic inflammation	[34, 38]	R&D Systems Luminex FCSTM08, 1:100
Cortisol, serum		Marker of systemic inflammation	[39]	Abcam AB108665, 1:2
Arg, serum	Arginine	Substrate for nitric oxide synthase	[40]	Aviva Systems OKEH02603, 1:2
SDMA, serum	Symmetric dimethylarginine	Inhibitor of nitric acid synthase, cardiac risk factor	[40, 41]	Abcam AB213973, neat
ADMA, serum	Asymmetric dimethylarginine	Inhibitor of nitric acid synthase, cardiac risk factor	[40, 41]	Novus NBP2-66,728, neat
TBARS, serum	Thiobarbituric acid reactive substances	Biproducts of lipid peroxidation, a measure of systemic inflammation	[40]	Abcam AB233471, 1:2
**Adipokines**				
Leptin, plasma heparin		Pro-inflammatory, high levels causing cartilage degeneration	[33]	R&D Systems Luminex FCSTM08 1:4
Resistin, plasma heparin		Upregulates synthesis of chemokines and cytokines by chondrocytes	[42]	R&D Systems Luminex FCSTM08 1:4
Adiponectin, plasma heparin		Pro-inflammatory, positively correlates with MMP-3 & COMP	[43]	R&D Systems Luminex FCSTM08 1:100
**Growth Factors**				
VEGF plasma heparin	Vascular Endothelial Growth Factor	Angiogenesis during osteophyte formation; positively corelated with OA radiographic severity	[44]	R&D Systems Luminex FCSTM09, 1:2
**Enzymes causing joint destruction**				
MMP-1, plasma heparin	Matrix metalloproteinase-1	A collagenase	[36]	R&D Systems Luminex FCSTM07, 1:5
MMP-3, plasma heparin	Matrix metalloproteinase-3	Hydrolysis of collagens and proteoglycans, activation of other MMPs	[36]	R&D Systems Luminex FCSTM07, 1:5
ADAMTS4, serum	A disintegrin and metalloproteinase with thrombospondin motifs-4	An aggrecanase	[45]	Novus NBP2-66,443, 1:200
ADAMTS5, serum	A disintegrin and metalloproteinase with thrombospondin motifs-5	An aggrecanase	[45]	Novus NBP2-66,444, Serum neat
**Molecule fragments resulting from joint destruction**				
CTX-1, urine	C-terminal telopeptide of type I collagen (bone destruction)	Detected in urine, normalized for creatinine concentration	[21, 46]	Novus NBP2-69,073 Urine neat
CTX-2, urine	C-terminal telopeptide of type II collagen (articular cartilage destruction)	Detected in urine, normalized for creatinine concentration	[21, 46]	Novus NBP2-76,435 Urine neat
C2C, urine	Collagenase-generated type II collagen cleavage epitope (articular cartilage destruction)	Detected in urine, normalized for creatinine concentration	[21, 47, 48]	MyBiosource MBS730283 Urine neat

### Joint health survey

Participants filled out a survey (Supplemental File) pertaining to joint health and details regarding their work. The survey included the Western Ontario and McMaster Universities Osteoarthritis Index (WOMAC) and was completed during both the pre-season and in-season sample collection visits. The in-season survey was timed to occur at the peak of the calving season for each respective rancher. The number of calvings personally performed by each participant at the in-season visit was reported, and because this visit was mid-season, it corresponded to approximately half the number of calvings each participant was expected to perform by season’s end. Participants were queried as to whether they employed a night calver, an individual responsible for performing calvings throughout the night, which allowed the rancher to sleep uninterrupted. No night calvers were included as participants. To quantify pain, the pre-season and in-season total WOMAC scores were tabulated. Additionally, the change in total WOMAC score was measured as the difference between in-season and pre-season (ΔWOMAC). Finally, the percent work still able to perform when limited by joint pain (“work capacity”) was also self-reported by each participant.

### Statistical analysis

One-way analysis of variance (ANOVA) assessed associations for statistical differences between quantitative mean biomarker levels from plasma, serum, and urine (the dependent variables) and the independent variables of the survey predictors (*e.g.,* number of calvings, etc.). All planned comparisons used an *a priori* significance level of α = 0.05.

Univariate ordinal logistic regression models for both pre- and in-season data were developed in Minitab to evaluate correlations between predictors and survey outcomes. Age, body mass index (BMI), number of calvings, night calver, and the change in total WOMAC were included as predictors, with total WOMAC score also being a main effect in the model for in-season responses. The joint health outcomes assessed by the model were total WOMAC score, work capacity, and days per month with joint pain. Model reduction was performed on each response where at least one significant predictor was identified. The *a priori* significance level was set at 0.05.

To identify potential subgroups of participants with similar results, ensemble clustering was performed in Python on pre-season, in-season, both seasons (“full study”), and seasonal difference (in-season – pre-season) of biomarker levels rancher subject IDs with various linkage functions and distance metrics [49]. The pre-season and in-season ensemble clusters only included their respective seasonal data.

Principal Component Analysis (PCA) determined distinct biomarkers of interest by inspection of PCA biplots for biomarkers with relatively large leverage within the dataset. To understand if these biomarkers varied between participant subsets, the distinct biomarkers were further examined by *post -hoc* analyses using one-way ANOVA with Tukey’s multiple-comparison at a 5% error rate. The independent variables were demographic and joint health predictors, with the distinct biomarker levels from each ensemble cluster as the dependent variables.

### Profiling

The statistically significant biomarkers determined by the *post -hoc* Tukey’s multiple-comparison tests were used to identify demographic similarities between subgroups of ranchers that corresponded to the statistically significant biomarker responses. These similarity profiles were used to characterize correlations between joint health predictors and biomarker levels in subgroups of ranchers.

## Results

Of 42 participants recruited, 3 were excluded due to having one or more exclusion criteria, and 1 participant was excluded because they did not complete the pre-season survey. Overall, participants had a mean age of 48.3 years (SD 12.3) and a mean BMI of 28.0 (SD 4.3); females (*n* = 10, 26.3%) averaged 49.6 years old (SD 10.4) and had a mean BMI of 28.2 (SD 4.9), while males (*n* = 28, 73.7%) averaged 47.8 years old (SD 13.0, one respondent did not provide an age) and had a mean BMI of 27.8 (SD 4.1).

**Fig. 2:**
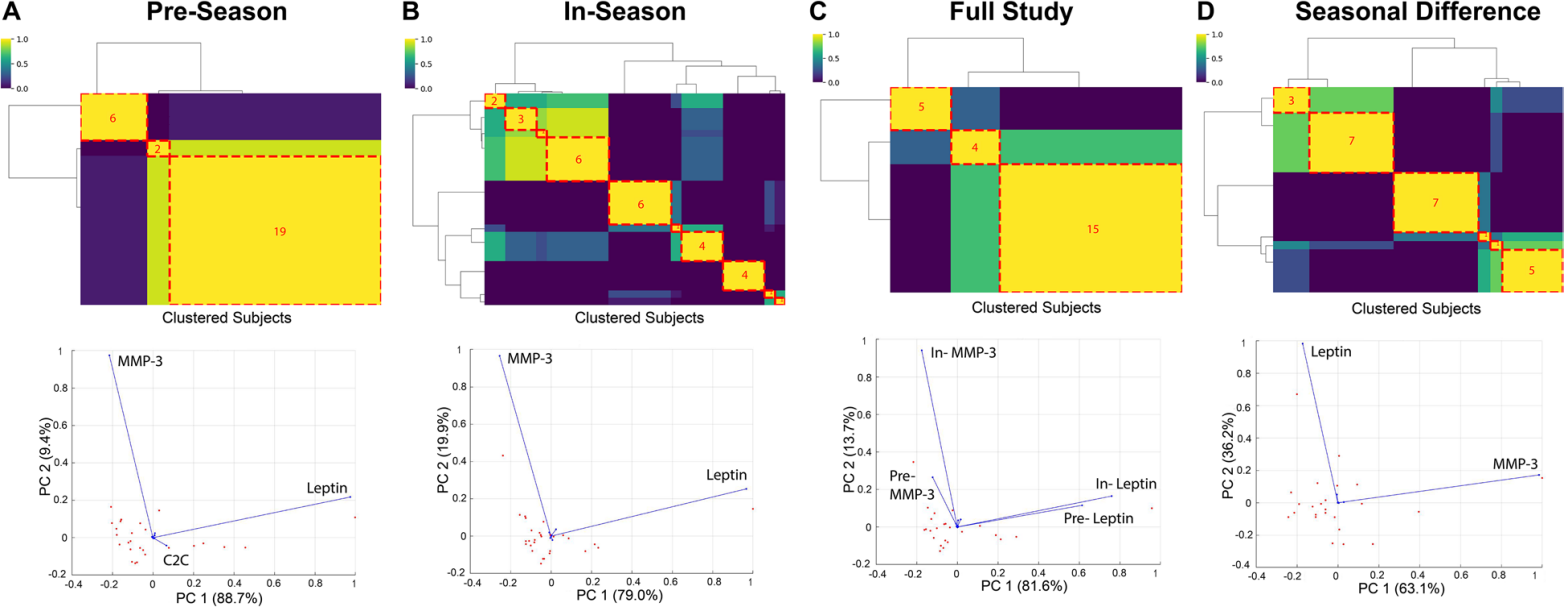
Ensemble clustergrams by subject (top) and principal component analysis (PCA) loading plots (bottom). **A** Pre-season, **B** In-season, **C** Full study, and **D** Seasonal difference (in—pre). Ensemble clustering was performed in Python for pre-season, in-season, both seasons (“full study”), and seasonal difference (in-season – pre-season) of biomarker levels and rancher subject IDs using the single, complete, average, and ward linkage functions with the Euclidean distance, and the single, complete, and average linkage functions with the squared-Euclidean, cosine and Chebyshev distance. PCA biplots were generated in MATLAB to identify biomarkers with relatively large leverage within the dataset (“distinct”) for profiling and *post-hoc* statistics. In-season biomarker responses were more distinct than pre-season, with rancher subjects clustering together in more unique subclusters during calving season. MMP-3 and leptin were distinct in all PCAs, while C2C was only distinct during pre-season

**Fig. 3:**
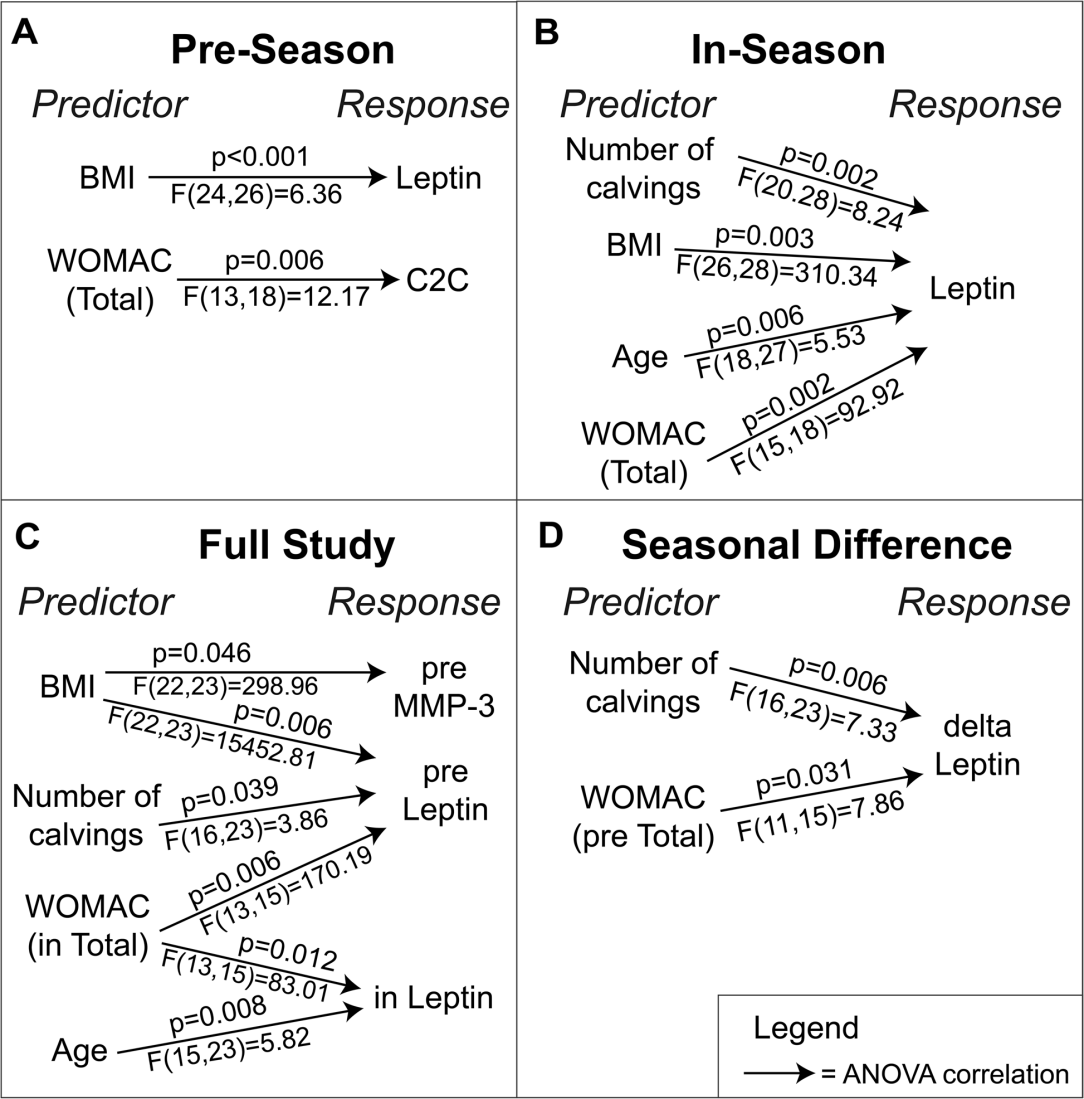
Distinct biomarker responses predicted by survey variables. **A** Pre-season, **B** In-season, **C** Full study, and **D** Seasonal difference (in – pre). One-way ANOVAs with Tukey’s multiple comparison were performed *post-hoc* in Minitab on distinct biomarkers identified by ensemble clustering and PCA. Minitab used adjusted p-values and f-values on a 0.05 significance level

Ensemble clustering identified distinct subgroups of participants in each comparison. For each subgroup identified, distinct differences between leptin and matrix metalloproteinase-3 (MMP-3) were found amongst the ranchers (Fig. 2). Additionally, the pre-season subgroup revealed distinct differences in C2C, while the seasonal difference comparison observed distinct differences in leptin and MMP-3 (Fig. 2). The seasonal difference in leptin and MMP-3 also switched principal component axes compared to the other seasonal subgroups (Fig. 2). One-way ANOVA with Tukey’s multiple comparison on the distinct biomarkers identified by ensemble clustering and PCA found that BMI was a significant predictor for leptin in pre-season, in-season, and “full-study” (utilizing both pre-season and in-season data) subgroups (Fig. 3). Total WOMAC was a significant predictor for C2C in the pre-season subgroups (Supplemental Figures, Fig. S1), for in-season leptin, for pre-season leptin in the full study subgroups, and for the pre-season component of the change in leptin from the seasonal difference subgroups (Fig. 3).

**Fig. 4:**
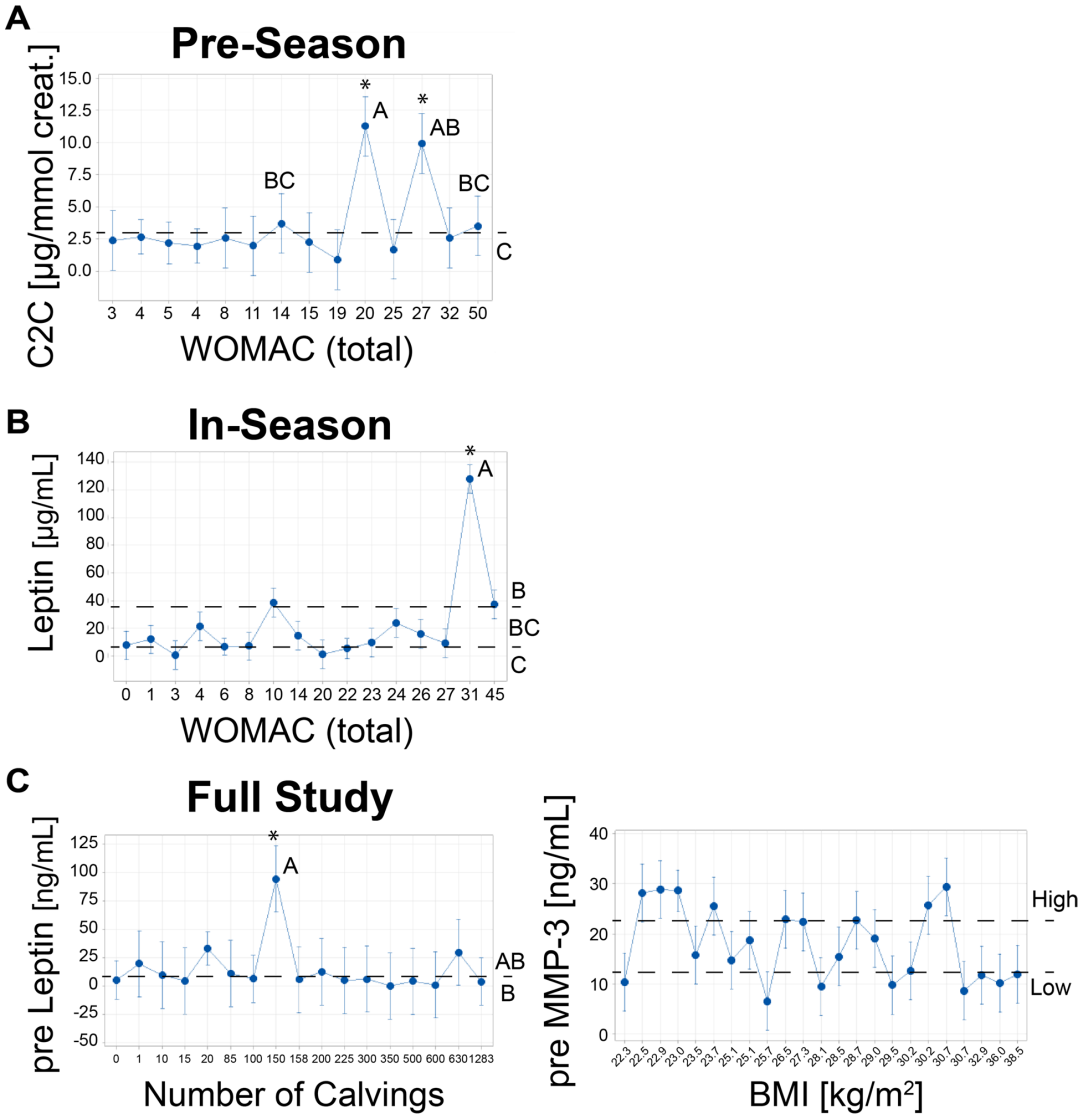
Sex-specific biomarker responses predicted by joint health outcomes and demographic information. **A** Pre-season, **B** In-season, and **C** Full study. Interval plots were produced in Minitab with 95% confidence intervals for the mean of each predictor level, pooled standard deviation for the intervals, and *post-hoc* ANOVA with Tukey’s group comparisons on a 0.05 significance level of adjusted p-values. Letters denote Tukey’s multiple comparison groups, *e.g.,* statistically significant differences between groups, such as A vs. B but not A vs. AB. A subset of females reporting higher WOMAC scores demonstrated statistically significant elevations to in-season leptin and pre-season C2C levels. In C), pre-season MMP-3 responses were rank ordered by intensity with the upper and lower third profiled due to split responses of BMI. Female ranchers in the lowest third for pre-season MMP-3 levels were also in the highest third for pre-season leptin levels, and the opposite was true for males. Leptin trends may inversely correlate with MMP-3

With profiling, three of the 38 subjects were distinct across multiple leptin and ensemble clustering subgroups; these subjects were all female, saw a decrease of in-season work capacity, had high WOMAC scores, and were heavily involved in their calving operations (Fig. 4, Table 3). In-season total WOMAC from the in-season timepoint identified that the same three subjects had significantly greater in-season leptin levels than a different group consisting of males with both light and heavy calving involvement plus a female rancher with low-involvement in calving (Fig. 4, Table 3). Within the full study ensemble comparison, pre-season MMP-3 and BMI showed two separate subgroups, where the group high in pre-season MMP-3 consisted entirely of male ranchers with mixed demographic and joint health data. The high MMP-3 male group featured the same subjects as the male group lowest in in-season leptin (Fig. 4, Supplemental Figures Fig. S2, and Table 3). The low pre-season MMP-3 group included the same three subjects that were elevated for in-season leptin (Fig. 4). For the seasonal difference ensemble comparison, two subjects had a large increase in leptin levels and experienced more than 19 days per month with joint pain during calving season compared to a group of subjects reporting fewer than 12 days per month with joint pain. The pre-season subgroup of C2C and total WOMAC indicated that two of the 38 ranchers had significantly greater pre-season C2C levels than all other ranchers (Fig. 4). Both were female, had a history of joint injury, high WOMAC scores, and had joints diagnosed with arthritis (Table 3).

**Table 3:** Demographic profiles of identified clusters of ranchers

Clustered rancher subject IDs	Biomarker trend vs. predictor	Number of subjects	Sex of subjects	Mean change work capacity (in – pre, % of max capacity)	Other demographic factor	Other biomarker interaction
13,34,36	High inLeptin vs total inWOMAC	3	0 M, 3F	-50	Highly involved in calving	3 of 3 low in preMMP-3
9,14,40,25, 30,4,38	Low inLeptin vs total inWOMAC	7	6 M, 1F	-6	Low calving activity (F), variable (M)^a^	
37,41,39,19,18, 15,4,38	High preMMP-3 vs BMI	8	8 M, 0F	-2	Variable^a^	8 of 8 low in preLeptin
34,11,29,12,13, 30,36,26	Low preMMP-3 vs BMI	8	3 M, 5F	-11	Variable^a^	5 of 8 high in at least one leptin comparison
13, 6	High preC2C vs total preWOMAC	2	0 M, 2F	-20	Arthritis diagnosis, recurring joint pain	

^a^Involvement in calving activity ranged from none or light (0–20 calvings performed by mid-season) to heavy (350 to 600 performed)

**Fig. 5:**
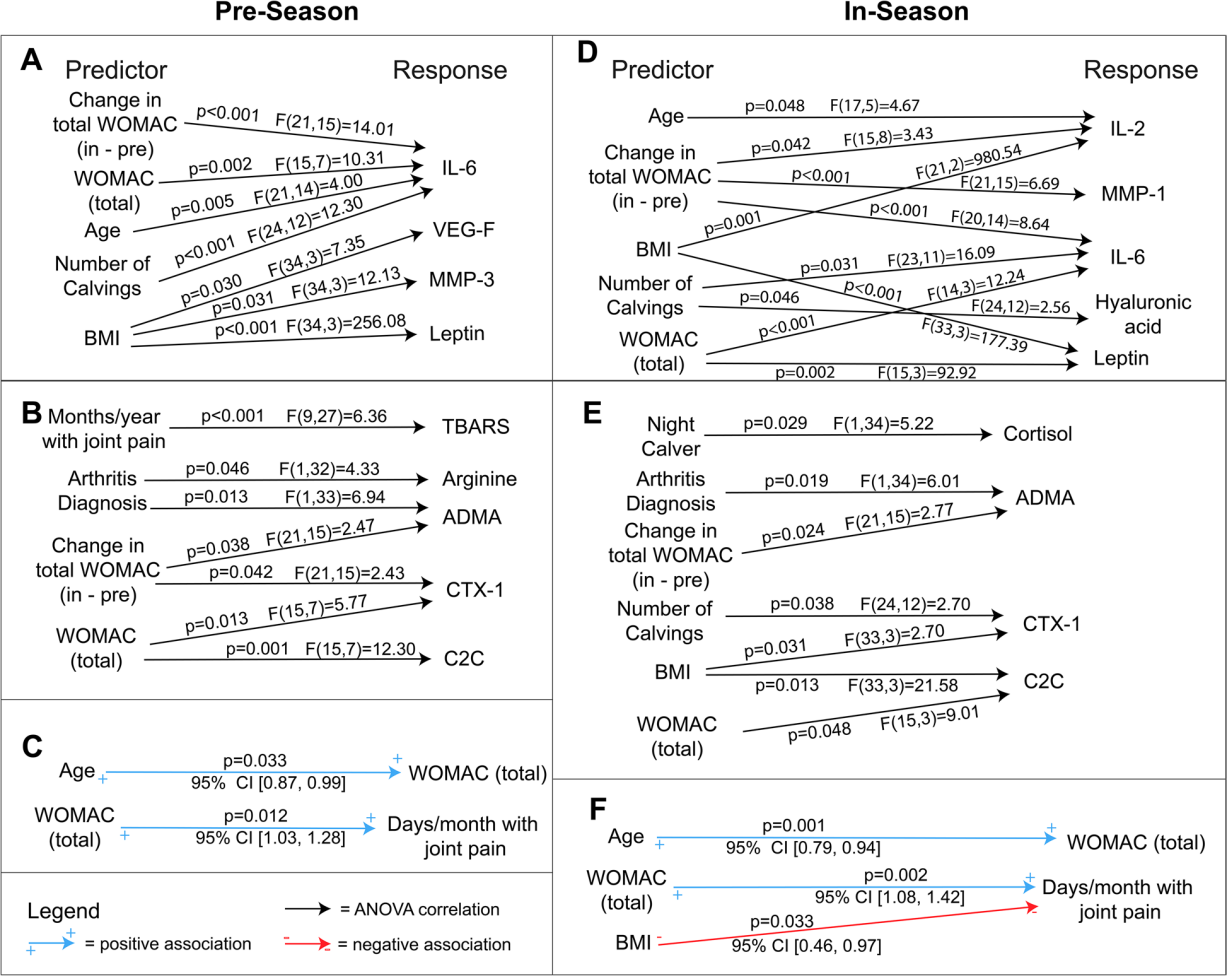
Significant predictors for seasonal biomarker and survey responses in a sample of ranchers. **A** Pre-season plasma biomarkers from one-way ANOVA, **B** Pre-season serum and urine biomarkers from one-way ANOVA, **C** Pre-season survey associations from ordinal logistic regression, **D** In-season plasma biomarkers from one-way ANOVA, **E** In-season serum and urine biomarkers form one-way ANOVA, and **F** In-season survey associations from ordinal logistic regression. One-way ANOVA and ordinal logistic regression were performed in Minitab on a 0.05 significance level

Analyses of additional OA-related biomarkers in blood and urine revealed that increased in-season total WOMAC score predicted elevated interleukin-6 (IL-6) levels in both seasons (Fig. 5). Total WOMAC and the number of calvings also predicted higher levels of IL-6 during both seasons, while age was only a predictor for pre-season. Increases in participant BMI values predicted elevations in VEGF and MMP-3. Elevated BMI and increases in in-season WOMAC score predicted higher levels of leptin. Total WOMAC scores were similarly associated with IL-6, number of days per month with joint pain, and in-season leptin. Hyaluronic acid (HA) levels were positively correlated with a greater number of calvings, and mean HA levels significantly increased in-season compared to pre-season (F(1,73) = 7.35, *p* = 0.008).

TBARS was elevated during in-season compared to pre-season and enhanced in ranchers experiencing more months per year with chronic joint pain during pre-season (F(9,27) = 6.26, *p* < 0.001). Cortisol was diminished during in-season compared to pre-season. In contrast, cortisol was elevated during calving season in ranchers who had a night calver compared to those who did not (F(1,34) = 5.22, *p* = 0.029). Increases of the in-season total WOMAC score and an arthritis diagnosis predicted elevated ADMA levels during both seasons (F(21,15) = 2.47, *p* = 0.038; F(1,33) = 6.94, *p* = 0.038). In contrast, arginine levels were reduced in ranchers diagnosed with arthritis during pre-season but elevated during in-season (F(1,32) = 4.33, *p* = 0.046). Increasing total WOMAC score predicted heightened levels of C2C during pre-season (F(15,7) = 12.30, p = 0.001). C2C was also enhanced by total WOMAC score and BMI during in-season (F(15,3) = 9.01, *p* = 0.048; F(33,3) = 21.58, p = 0.013). During pre-season, total WOMAC score and a greater increase in in-season total WOMAC relative to pre-season total WOMAC predicted elevated CTX-1 levels (F(15,7) = 5.77, *p* = 0.013; F(21,15) = 2.43, p = 0.042). Moreover, greater BMI and number of calvings predicted elevated CTX-1 levels during in-season (F(33,3) = 2.70, *p* = 0.031; F(24,12) = 2.70, *p* = 0.038). The following biomarkers were not found to be statistically significant by one-way ANOVA nor ordinal logistic regression against the predictors assessed: IL-1ra, IL-4, IL-8, MCP-1 (CCL2), resistin, adiponectin, CRP, SDMA, ADAMTS4, ADAMTS5, and CTX-II.

Total WOMAC score positively associated with the number of days per month with joint pain during pre-season (95% CI [1.03, 1.28], p = 0.012) and in-season (95% CI [1.08, 1.42], *p* = 0.002) while joint pain was negatively associated with BMI during in-season (95% CI [0.46, 0.97], *p* = 0.033) (Fig. 5). Age positively associated with pre-season total WOMAC (95% CI [0.87, 0.99], *p* = 0.033) and in-season total WOMAC (95% CI [0.79, 0.94], *p* = 0.001) (Fig. 5).

Arthritis diagnosis correlated with a statistically significant reduction in pre-season work capacity compared to no arthritis as determined by one-way ANOVA (F(1,32) = 5.28), *p* = 0.028) but not for in-season.

## Discussion

Our study sought to determine whether calving season is an independent risk factor for the development of OA in Montana ranchers. Calving operations have been identified by ranchers as the most physically demanding season of the year and has features that may contribute to joint injury and physiologic stress [8]. Pre-season and within calving season, we assayed blood, urine and saliva samples for biomarkers known to be associated with OA and with increasing OA severity and analyzed these data with regard to predictors included in our pre-season and in-season survey. An increased number of calvings performed was associated with elevations of IL-6, HA and CTX-1, providing evidence of an association between calving season and OA pathogenesis. Serum HA concentration has been shown to correlate with the number of joints having OA and is predictive of knee OA progression [37, 50]. Urinary CTX-1 was also positively correlated with increased BMI in our study. CTX-I, a type I collagen C-terminal molecular fragment derived from subchondral bone destruction and resorption in OA, was significantly predictive of OA pain and radiographic progression over both one and two years in a prospective study [21].

Regarding articular cartilage enzymatic destruction, our study did not show elevations of urinary CTX-II. This is derived from cleavage of type II collagen in articular cartilage and positively correlates with knee OA severity and the concurrent presence of OA [21, 46]. However, in our study, urinary C2C epitope, another articular cartilage type II collagen cleavage product, showed a positive association with BMI in-season (*p* = 0.013) and was significantly predicted by increasing WOMAC score. C2C was particularly elevated in two female ranchers having a history of joint injury.

The association in our study between BMI and in-season urinary CTX-1 and C2C suggests a vulnerability of overweight or obese ranchers to enzymatic joint damage. Multiple mechanisms could be responsible for this observation. Increased adiposity is associated with higher leptin levels, and weight loss lowers leptin levels, and leptin levels are positively correlated with the presence of knee and hip OA [33, 51, 52]. Leptin promotes the intra-articular synthesis of inflammatory mediators and metalloproteinases (MMPs) [53]. Mechanical overloading in overweight or obesity, combined with the rigors of calving operations, may represent another mechanism, as synovial fluid C2C levels are elevated after knee injury, considered to be MMP-mediated [47]. Urinary C2C is also positively correlated with OA grade [54].

The linkage between increased number of calvings, which relates to workload, and IL-6 may be due to the characteristics of calving season reported in a previous study, such as high levels of physical exertion, lack of sleep, cold weather, and a nutritionally poor diet [8]. These characteristics likely impact systemic inflammation and influence the profile of circulating metabolic and immune/inflammatory-signaling mediators. Indeed, TBARS, a surrogate marker of lipid peroxidation, showed higher in-season than pre-season levels (*p* < 0.001) [40]. IL-6 can be elevated in the blood of OA patients[37, 50], but it is a pleiotropic cytokine also produced by contracting skeletal muscle [55, 56]. Thus, the observed correlation between IL-6 and workload may reflect an acute response to physical exercise rather than a damaging effect of calving operations, but a positive association between WOMAC score and ADMA concentrations was also observed, as well as between WOMAC and increases in IL-2, IL-6, MMP-1, leptin, and CTX-1. ADMA is a risk factor for rheumatoid arthritis and disease duration and is pro-inflammatory [55]. ADMA functions as an inhibitor of nitric acid synthase, competing with arginine for site binding [56, 57]. Overall, these results point to a correlation between joint health symptoms during calving season and alterations in markers of immune signaling, inflammatory signaling, oxidative stress, and collagen degradation.

Total WOMAC score, number of calvings, and change in WOMAC were the main calving-oriented predictors for seasonal modulations in OA-related biomarker levels and joint health responses. This demonstrates a tripartite link between workload, symptoms, and OA biomarker levels. Associations between increasing WOMAC and work capacity agreed with prior findings of declining productivity with high WOMAC score and arthritis diagnosis [8].

Using ensemble clustering and PCA analysis, we found evidence of sexual dimorphism in rancher biomarkers, with elevated leptin and decreased MMP-3 in female ranchers. In male ranchers, leptin decreased and MMP-3 was elevated. In-season biomarker responses were more distinct amongst the ranchers than pre-season, with additional unique subclusters observed. Elevated levels of MMP-3 have been evaluated as a prognostic marker for the progression of cartilage damage for patients with knee OA [58]. MMP-3, leptin, and C2C represent OA biomarkers relating to enzymatic processes responsible for tissue degradation, endocrine molecules with systemic metabolic status, and byproducts of joint destruction, respectively. Leptin levels are positively associated with OA severity [33], which may promote production of inflammatory mediators by chondrocytes and other joint cells that have been implicated as a systemic factor in the progression of hip and knee OA [59].

Both serum and synovial fluid leptin concentrations are higher in obese patients, and its detrimental effects on joint tissues are enhanced in obese OA patients. Leptin concentration in synovial fluid is increased and the intra-articular negative regulators of leptin sOb-R (soluble leptin receptor) and SOCS-3 (suppressor of cytokine signaling-3) are diminished in obesity, leading to increased stimulation by leptin of matrix MMP-1, MMP-3, IL-6 and NO production by chondrocytes [53]. Leptin has been proposed as a metabolic mediator in the heightened risk for OA in adult women [60, 61]. Previously, sexual dimorphism in plasma leptin concentrations has been found independent of adipose deposits, suggesting hormonal mediation driving elevated circulating leptin in pre-menopausal women compared to men [62]. Furthermore, local leptin production by non-adipose cells, osteoblasts and chondrocytes, has been hypothesized to contribute to heightened leptin levels observed in cartilage and osteophytes of people diagnosed with OA [61].

In our study, leptin may underlie the observed sex-specific trends found in the subset of female ranchers, whose leptin levels may be more sensitive to calving workload intensity than those of male ranchers. Male ranchers showing low in-season leptin reported both high and low WOMAC scores and varied workload. In contrast significantly higher in-season leptin was detected only for female ranchers with a high workload.

Although elevated leptin concentrations can enhance MMP-1 and MMP-3 levels in the synovial fluid of OA patients [63], in-season or seasonal changes in MMP-3 were not significant by *post -hoc* ANOVA in this study. In-season leptin trends inversely related to pre-season MMP-3 levels during profiling, with MMP-3 elevated in males before rather than during calving season. This suggests that calving may have been a better predictor for suppressing distinctions between the male ranchers’ MMP-3 responses, while leptin was more closely related to OA structural deterioration and OA severity than calving activity. For the change of seasonal leptin against the number of calvings, ranchers with the least change in leptin levels also reported the lowest joint pain. BMI was only surveyed at a single time point and adiposity data were not collected, so a comparison of biomarker responses in the full study or between sexes could not be determined by these metrics.

Regarding the sexual dimorphism of OA, the three female ranchers with recurring significantly different biomarker responses each disclosed a history of joint injury or arthritis diagnosis, even if their reported pain was minor. Knee, hip, and hand OA are more prevalent in women, especially around menopause [64]. Sexual dimorphism in OA also affects bone microstructure and mechanical properties [65]. Increasing the number of female ranchers recruited in this study and exploring cartilage and bone pathophysiology would help characterize how strongly sex was associated with the observed differences in biomarker levels compared to the joint factors that are known to influence bone and cartilage microstructure.

A history of heavy occupational work has been identified alongside male sex as a protective factor against OA, while BMI has been associated with higher risk for OA [66]. Similar studies on OA risk and occupation found associations between physically-demanding occupational activities and increased odds of knee OA compared to lower odds of knee OA for sedentary workers [67]. Physically demanding work included occupations in agriculture like farming and industrial-construction sectors such as metal working and construction, which feature heavy lifting, climbing, kneeling, and squatting [67]. This description resembles the manual labor described in rancher focus groups, with heightened labor intensity during calving season, suggesting that broader translation of our findings in a sample of ranchers may be possible beyond the rancher population. Determining the decisive factors that contribute to OA protection rather than OA progression will support improved clinical guidelines for OA prevention and mitigation, not only for ranchers, but also for other workers in the agricultural, industrial, and construction sectors.

The limitations of our study include an inability to collect saliva upon the subjects’ morning awakening, for cortisol analysis. This is desirable because of the diurnal rhythm of this hormone [68] but was impossible due to the unpredictable timing of calvings and ranchers’ schedules. This complicated the determination of whether ranchers with night calvers might demonstrate reduced systemic inflammation, due to improved sleep. Cortisol concentrations may also have been influenced by the fact that ranchers with chewing tobacco were asked to first swish and spit with water, perhaps diluting the saliva subsequently collected. Tobacco chewers constituted about half of our male subjects and none of our female subjects. HA concentrations are elevated after meals, making pre-prandial collection desirable, but meal schedules are substantially disrupted during calving [8]. While our study showed strong associations between certain OA-related biomarkers and calving season, the source of these circulating biomarkers is unknown. Joint damage, systemic oxidative stress, and excess adipose tissue in a cohort having a mean BMI in the overweight category are all viable candidates.

## Conclusion

In conclusion, we found significant associations between OA-related biomarkers and calving season factors that are OA risk factors. Biomarkers in this sample of ranchers were associated with metabolic and immune signaling, oxidative stress, and molecular fragments from tissue destruction. We found a linkage between workload during calving season and OA risk, particularly with regard to elevations of plasma HA and urinary CTX-I, both of which are predictive of OA progression, the former also being positively associated with the number of joints having OA [21, 37].

This study found compelling evidence for sexual dimorphism in the biomarker responses of leptin and MMP-3. Leptin especially had a strong influence on biomarker responses in rancher profiles or associations with joint health predictors in women. These findings suggest that female ranchers were more sensitive to the physically demanding labor during calving season when they had a history of joint injury, arthritis diagnosis, and high WOMAC score. MMP-3 levels were more enhanced in male ranchers in pre-season than their female counterparts. These sex-specific responses to calving operations impacted OA biomarkers and may influence sex-specific pathophysiology.

Identifying characteristic biomarkers and joint responses in ranchers during calving season may present opportunities for therapeutic intervention. These advances may prevent productivity losses and support improved joint health in agricultural workers who are susceptible to OA.

## Supplementary Information

**Additional file 1: Supplemental file S1.**Pre-season Joint Symptoms Survey.

**Additional file 2: Fig. S1.** Pre-season biomarker responses predicted by joint health outcomes and demographic information. Interval plots were produced in Minitab with 95% confidence intervals for the mean of each predictor level, pooled standard deviation for the intervals, and post-hoc ANOVA with Tukey’s group comparisons on a 0.05 significance level of adjusted p-values. Night calver (y=yes, n=no, *=no response) and days per month with joint pain (1=1-6, 2=7-12, 3=13-18, 4=19-24, 5=25-all). **Fig. S2.** In-season biomarker responses predicted by joint health outcomes and demographic information. Interval plots were produced in Minitab with 95% confidence intervals for the mean of each predictor level, pooled standard deviation for the intervals, and *post-hoc* ANOVA with Tukey’s group comparisons on a 0.05 significance level of adjusted p-values. Night calver (y=yes, n=no, *=no response) and days per month with joint pain (1=1-6, 2=7-12, 3=13-18, 4=19-24, 5=25-all). **Fig. S3.** Combination biomarker responses predicted by joint health outcomes and demographic information. Interval plots were produced in Minitab with 95% confidence intervals for the mean of each predictor level, pooled standard deviation for the intervals, and *post-hoc* ANOVA with Tukey’s group comparisons on a 0.05 significance level of adjusted p-values. Night calver (y=yes, n=no, *=no response) and days per month with joint pain (1=1-6, 2=7-12, 3=13-18, 4=19-24, 5=25-all). **Fig. S4.** Seasonal difference biomarker responses predicted by joint health outcomes and demographic information. Interval plots were produced in Minitab with 95% confidence intervals for the mean of each predictor level, pooled standard deviation for the intervals, and *post-hoc* ANOVA with Tukey’s group comparisons on a 0.05 significance level of adjusted p-values. Night calver (y=yes, n=no, *=no response) and days per month with joint pain (1=1-6, 2=7-12, 3=13-18, 4=19-24, 5=25-all).

## Data Availability

The survey is available as a supplemental file. Summary data are provided in the manuscript in both tabular and graphical form.

## Abbreviations

OA: Osteoarthritis
WOMAC: Western Ontario and McMaster Universities Arthritis Index
RR: Relative risk
COMP: Collagen oligomeric matrix protein
CTX-II: C-terminal telopeptide of collagen type II
MSU: Montana State University
EDTA: Ethylenediaminetetraacetic acid
HERB: Health, Education, and Research Bus
K-L: Kellgren-Lawrence
ΔWOMAC: Change in total WOMAC score
ANOVA: Analysis of variance
BMI: Body mass index
PCA: Principal Component Analysis
MMP-3: Matrix metalloproteinase-3
C2C: Cleavage of type II collagen epitope
IL-6: Interleukin-6
VEGF: Vascular endothelial growth factor
HA: Hyaluronic acid
TBARS: Thiobarbituric acid reactive substances
ADMA: Asymmetric dimethylarginine
CTX-I: C-terminal telopeptide of collagen type I
IL-1ra: Interleukin 1 receptor antagonist
IL-4: Interleukin-4
IL-8: Interleukin-6
MCP-1: Monocyte chemoattractant protein-1
CCL2: C–C motif ligand 2
CRP: C-reactive protein
SDMA: Symmetric dimethylarginine
ADAMTS4: A disintegrin and metallopeptidase with thrombospondin type 1 motif 4
ADAMTS5: A disintegrin and metallopeptidase with thrombospondin type 1 motif 5
sObR: Soluble leptin receptor
SOCS-3: Suppressor of cytokine signaling-3
MMP-1: Matrix metalloproteinase-1
NOS: Nitric oxide synthase
