# Supplementary material for: Sex-specific effects of calving season on joint health and biomarkers in Montana ranchers
Source: BMC Musculoskelet Disord. 2023 Jan 31;24:80. doi: 10.1186/s12891-022-05979-2 (PMC9887842; doi:10.1186/s12891-022-05979-2)
Supplement: Supplementary file 1 — Additional file 1: Supplemental file S1.Pre-season Joint Symptoms Survey. [file 12891_2022_5979_MOESM1_ESM.docx]

Appendix 1

Joint Symptoms Survey

All responses are confidential

# Please list your age

# How much do you weigh?

# How tall are you? ft. in.

# About how many calvings have you performed or attended this season?

# Do you have a night calver? Yes No

# Have you ever had a joint injury? Yes No

# What joint(s) was (were) injured (check all that apply):

# shoulder elbow wrist hand or fingers

# neck (cervical spine) mid- or low-back

# hip knee ankle foot or toes

# Did a joint injury cause you to miss work, school or sports? Yes No

# Do you have pain in any joints at any time during the year? Yes No

# If no, skip to question 17

# During how many months of the year are there at least some days with joint pain?

# During how many days of each month do you have joint pain?

# 1 to 6 7-12 13-18 19-24 25 to all days

# What are your most painful agricultural activities?

# Calving operations Feeding livestock Lifting Carrying Climbing

# Branding operations Riding horseback Walking Driving tractor

# Driving 4-wheeler

# Has a doctor (or other health care provider) diagnosed you with arthritis? Yes No

# If you have arthritis, which type? osteoarthritis rheumatoid arthritis gout

# psoriatic (psoriasis-related) lupus Lyme arthritis

# other: not sure

# Did a doctor obtain x-rays of your painful joint(s)? yes no

# In which joint(s) have you been diagnosed with arthritis? (check all that apply) none

# knee hip shoulder neck (cervical spine)

# low back (lumbar spine) wrist or hand ankle or foot

# elbow other joint:

# In which joint(s) do you have pain but have not been diagnosed with arthritis? (check all that apply) not applicable

# knee hip shoulder neck (cervical spine)

# low back (lumbar spine) wrist or hand ankle or foot

# elbow other joint:

# Have you had a joint replacement? (check all that apply) never had a joint replacement

# knee hip shoulder wrist or hand

# ankle or foot other:

# Have you had spine surgery? Yes No

# If you have joint pain, does it limit your ability to do your work? (skip if you have no joint pain)

# Yes No

# If your physical work is limited by joint pain, to what extent? Please mark on the line the percentage of your physical work you are still able to do.

# not applicable – I have no joint pain and am not limited in any way.

# No physical work half of my work All physical work

# If you have no pain in your hips or knees, you are done with this survey. Thank you for participating.

# Only if you have joint pain in your hips or knees, please fill out the following standardized Western Ontario and McMaster University (WOMAC) survey. Please check only one box for each question:

# Think about the pain you felt in your hip or knee in the past 48 hours. How much pain did you have?

|  | none | mild | moderate | severe | extreme |
| --- | --- | --- | --- | --- | --- |
| Walking on a flat surface |  |  |  |  |  |
| Going up and down stairs |  |  |  |  |  |
| At night while in bed, pain disturbs your sleep |  |  |  |  |  |
| Sitting or lying down |  |  |  |  |  |
| Standing upright |  |  |  |  |  |
|  |  |  |  |  |  |

# WOMAC pain score:

# Think about the stiffness (not pain) you have in your hip or knee in the past 48 hours. Stiffness is a sensation of decreased ease in moving your joint.

|  | none | mild | moderate | severe | extreme |
| --- | --- | --- | --- | --- | --- |
| How severe is your stiffness after first awakening in the morning? |  |  |  |  |  |
| How severe is your stiffness after sitting, lying down, or resting during the day? |  |  |  |  |  |
|  |  |  |  |  |  |

# WOMAC stiffness score:

# Think about the difficulty you have had in doing the following daily physical activities due to your hip or knee during the past 48 hours. By this we mean your ability to move around and look after yourself. What degree of difficulty do you have?

|  | none | mild | moderate | severe | extreme |
| --- | --- | --- | --- | --- | --- |
| Descending stairs |  |  |  |  |  |
| Ascending stairs |  |  |  |  |  |
| Rising from sitting |  |  |  |  |  |
| Standing |  |  |  |  |  |
| Bending to the floor |  |  |  |  |  |
| Walking on flat surfaces |  |  |  |  |  |
| Getting in or out of a car, or on or off a bus |  |  |  |  |  |
| Going shopping |  |  |  |  |  |
| Putting on your socks or stockings |  |  |  |  |  |
| Rising from the bed |  |  |  |  |  |
| Taking off your socks or stockings |  |  |  |  |  |
| Lying in bed |  |  |  |  |  |
| Getting in or out of the bath |  |  |  |  |  |
| Sitting |  |  |  |  |  |
| Getting on or off the toilet |  |  |  |  |  |
| Performance of heavy domestic duties |  |  |  |  |  |
| Performance of light domestic duties |  |  |  |  |  |
|  |  |  |  |  |  |

# WOMAC functional score:

# Total WOMAC score:
