# Supplementary material for: Sex-specific effects of calving season on joint health and biomarkers in Montana ranchers
Source: BMC Musculoskelet Disord. 2023 Jan 31;24:80. doi: 10.1186/s12891-022-05979-2 (PMC9887842; doi:10.1186/s12891-022-05979-2)
Supplement: Supplementary file 2 — Additional file 2: Fig. S1. Pre-season biomarker responses predicted by joint health outcomes and demographic information. Interval plots were produced in Minitab with 95% confidence intervals for the mean of each predictor level, pooled standard deviation for the intervals, and post-hoc ANOVA with Tukey’s group comparisons on a 0.05 significance level of adjusted p-values. Night calver (y=yes, n=no, *=no response) and days per month with joint pain (1=1-6, 2=7-12, 3=13-18, 4=19-24, 5=25-all). Fig. S2. In-season biomarker responses predicted by joint health outcomes and demographic information. Interval plots were produced in Minitab with 95% confidence intervals for the mean of each predictor level, pooled standard deviation for the intervals, and post-hoc ANOVA with Tukey’s group comparisons on a 0.05 significance level of adjusted p-values. Night calver (y=yes, n=no, *=no response) and days per month with joint pain (1=1-6, 2=7-12, 3=13-18, 4=19-24, 5=25-all). Fig. S3. Combination biomarker responses predicted by joint health outcomes and demographic information. Interval plots were produced in Minitab with 95% confidence intervals for the mean of each predictor level, pooled standard deviation for the intervals, and post-hoc ANOVA with Tukey’s group comparisons on a 0.05 significance level of adjusted p-values. Night calver (y=yes, n=no, *=no response) and days per month with joint pain (1=1-6, 2=7-12, 3=13-18, 4=19-24, 5=25-all). Fig. S4. Seasonal difference biomarker responses predicted by joint health outcomes and demographic information. Interval plots were produced in Minitab with 95% confidence intervals for the mean of each predictor level, pooled standard deviation for the intervals, and post-hoc ANOVA with Tukey’s group comparisons on a 0.05 significance level of adjusted p-values. Night calver (y=yes, n=no, *=no response) and days per month with joint pain (1=1-6, 2=7-12, 3=13-18, 4=19-24, 5=25-all). [file 12891_2022_5979_MOESM2_ESM.pdf]

Supplemental Information

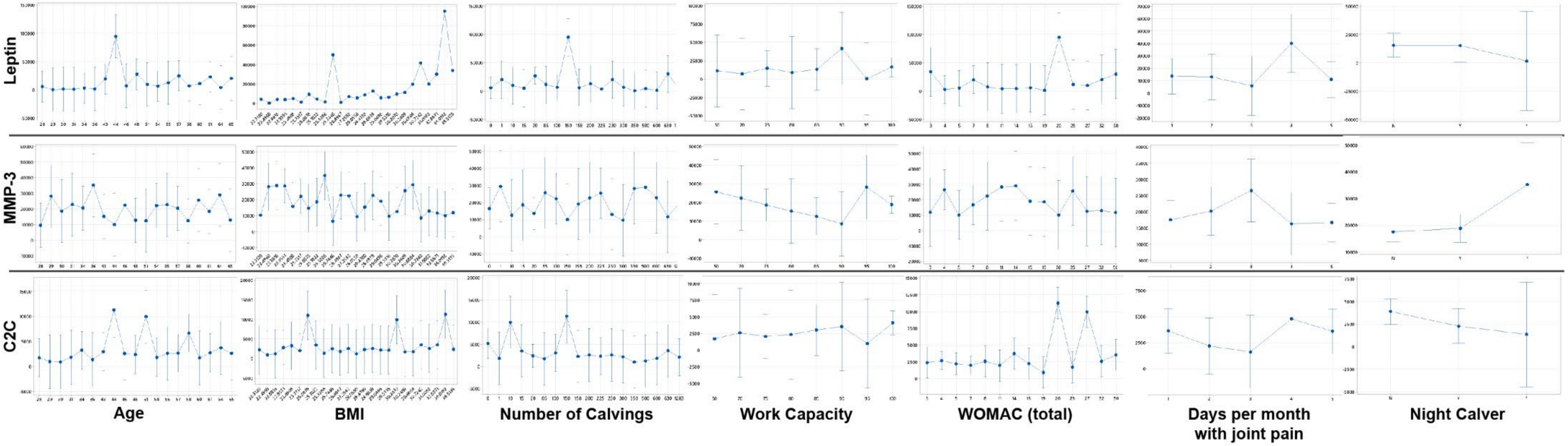

Fig. S1: Pre-season biomarker responses predicted by joint health outcomes and demographic information. Interval plots were produced in Minitab with 95% confidence intervals for the mean of each predictor level, pooled standard deviation for the intervals, and *post-hoc* ANOVA with Tukey's group comparisons on a 0.05 significance level of adjusted p-values. Night calver (y=yes, n=no, *=no response) and days per month with joint pain (1=1-6, 2=7-12, 3=13-18, 4=19-24, 5=25-all).

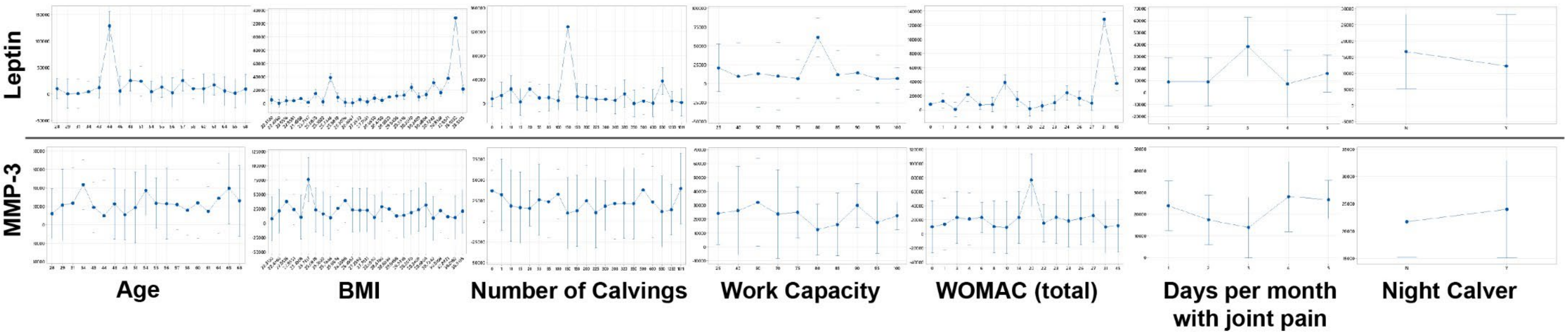

Fig. S2: In-season biomarker responses predicted by joint health outcomes and demographic information. Interval plots were produced in Minitab with 95% confidence intervals for the mean of each predictor level, pooled standard deviation for the intervals, and *post-hoc* ANOVA with Tukey's group comparisons on a 0.05 significance level of adjusted p-values. Night calver (y=yes, n=no, *=no response) and days per month with joint pain (1=1-6, 2=7-12, 3=13-18, 4=19-24, 5=25-all).

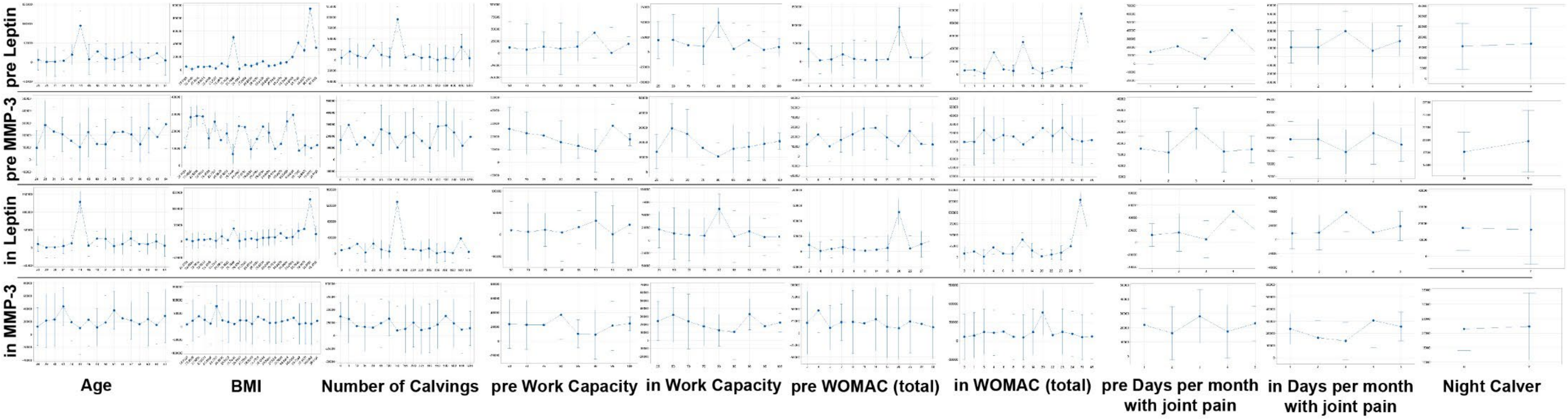

Fig. S3: Combination biomarker responses predicted by joint health outcomes and demographic information. Interval plots were produced in Minitab with 95% confidence intervals for the mean of each predictor level, pooled standard deviation for the intervals, and *post-hoc* ANOVA with Tukey's group comparisons on a 0.05 significance level of adjusted p-values. Night calver (y=yes, n=no, *=no response) and days per month with joint pain (1=1-6, 2=7-12, 3=13-18, 4=19-24, 5=25-all).

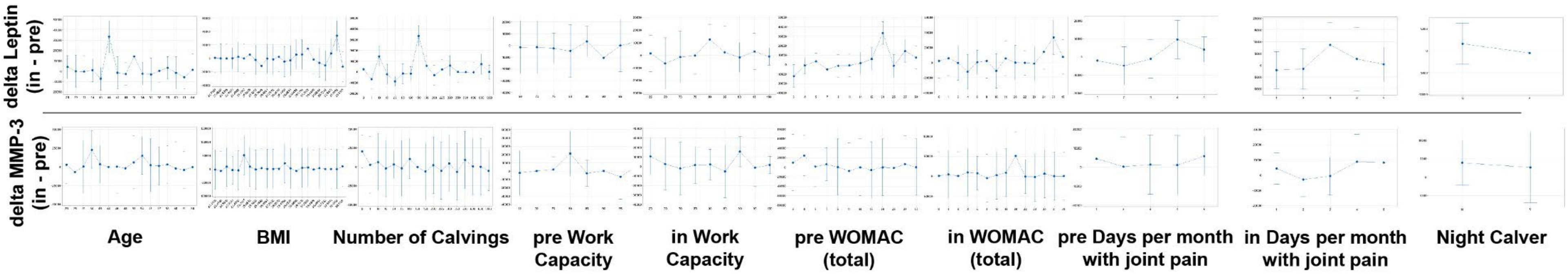

Fig. S4: Seasonal difference biomarker responses predicted by joint health outcomes and demographic information. Interval plots were produced in Minitab with 95% confidence intervals for the mean of each predictor level, pooled standard deviation for the intervals, and *post-hoc* ANOVA with Tukey's group comparisons on a 0.05 significance level of adjusted p-values. Night calver (y=yes, n=no, *=no response) and days per month with joint pain (1=1-6, 2=7-12, 3=13-18, 4=19-24, 5=25-all).
